# Early evidence of natal‐habitat preference: Juvenile loons feed on natal‐like lakes after fledging

**DOI:** 10.1002/ece3.7134

**Published:** 2020-12-28

**Authors:** Brian A. Hoover, Kristin M. Brunk, Gabriella Jukkala, Nathan Banfield, Andrew L. Rypel, Walter H. Piper

**Affiliations:** ^1^ Schmid College of Science and Technology Chapman University Orange CA USA; ^2^ Department of Forest and Wildlife Ecology University of Wisconsin‐Madison Madison WI USA; ^3^ Department of Natural Resources University of Illinois Champaign IL USA; ^4^ 8 Rainbow Circle Montgomery City MO USA; ^5^ Department of Wildlife, Fish, and Conservation Biology University of California Davis Davis CA USA

**Keywords:** habitat selection, juveniles, loons, natal habitat preference induction, NHPI

## Abstract

Many species show natal habitat preference induction (NHPI), a behavior in which young adults select habitats similar to those in which they were raised. However, we know little about how NHPI develops in natural systems. Here, we tested for NHPI in juvenile common loons (*Gavia immer*) that foraged on lakes in the vicinity of their natal lake after fledging. Juveniles visited lakes similar in pH to their natal lakes, and this significant effect persisted after controlling for spatial autocorrelation. On the other hand, juveniles showed no preference for foraging lakes of similar size to their natal one. When lakes were assigned to discrete classes based on size, depth, visibility, and trophic complexity, both juveniles from large lakes and small lakes preferred to visit large, trophically diverse lakes, which contained abundant food. Our results contrast with earlier findings, which show strict preference for lakes similar in size to the natal lake among young adults seeking to settle on a breeding lake. We suggest that NHPI is relaxed for juveniles, presumably because they select lakes that optimize short‐term survival and growth. By characterizing NHPI during a poorly studied life stage, this study illustrates that NHPI can take different forms at different life stages.

## Graphical abstract

Many species have been shown to engage in natal‐habitat‐preference induction (NHPI), the behavior in which young adults recruit into territories with similar physical characteristics as the one in which they were raised. Here, we explored the ontogeny of this behavior by showing that juvenile loons exhibit NHPI when they explore nearby lakes for food, yet use different habitat criteria than do adults that express NHPI. Thus, the significant results here show that NHPI effects vary with age and proximate drivers (breeding habitat vs. foraging habitat).

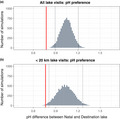

## 1 INTRODUCTION

Settling in appropriate habitat is a significant and critical challenge for young animals (Becker & Bradley, 2007; Greenwood, 1980). This challenge is not trivial; most natural environments are temporally and spatially variable, so individuals must seek out habitats that enhance fitness and avoid habitats that do not (Clark & Shutler, 1999; Orians & Wittenberger, 1991). Fitness‐enhancing habitats include those with ample food resources (Misenhelter & Rotenberry, 2000), few predators or environmental stressors (Eggers et al., 2005; Karr & Freemark, 1983), and consistent reproductive success (Boulinier et al., 2002; Switzer, 1997).

Fretwell and Lucas (1969) developed a set of influential models under the simplifying assumption that all individuals within a species assessed habitats similarly. They envisioned two scenarios for settlement under this assumption: (a) animals should distribute themselves in proportion to available resources (e.g., ideal free distribution; Fretwell and Lucas (1969)), or, (b) dominant individuals would secure the highest quality spaces, forcing poorer competitors to occupy inferior spaces (e.g., an ideal despotic distribution; Fretwell (1972)). While useful conceptually, these models predict habitat settlement imperfectly in many vertebrates, as prior knowledge of spaces (i.e., familiarity), previous experience, and phenotypic differences often cause individuals to favor certain habitat types over others (Piper, 2011).

One clear habitat settlement strategy is natal habitat preference induction (NHPI), the tendency of young adult settlers to select breeding habitats that are similar to those in which they were raised (Davis & Stamps, 2004; Stamps & Davis, 2006). Under this model, the experiences of a juvenile help inform later recruitment decisions regarding the qualities of future habitat (Stamps & Davis, 2006). Specifically, favorable experiences in a specific natal habitat increase the likelihood of a young animal recruiting into that habitat (Stamps et al., 2009) and provide energetic and fitness benefits (Davis & Stamps, 2004; Stamps, 2001; Stamps & Davis, 2006). “Habitat cuing” might allow a young settler to identify an acceptable habitat and avoid habitats of low quality (Davis, 2008; Davis & Stamps, 2004). Alternatively, “habitat training” suggests that developmental plasticity produces behavioral specializations that allow an individual to exploit a particular habitat type effectively throughout life (Davis & Stamps, 2004; Stamps et al., 2009).

Previous studies have documented NHPI within natural populations in insects (Davis, 2008), fishes (Dixson et al., 2014), birds (Mannan et al., 2007; Piper et al., 2013), and mammals (Karlin & Chadwick, 2012; Mabry & Stamps, 2008; Merrick & Koprowski, 2016), yet no studies to our knowledge have investigated the ontogeny of this process prior to recruitment into adult territories. Thus, among species that inhabit the same environment across developmental life stages (e.g., species without larval dispersal), there is a conceptual gap in our knowledge of how NHPI behavior changes with life stage. Specifically, we do not know whether NHPI is expressed more strongly in juveniles than in adults, nor whether habitats are assessed differently at different life stages. These questions are difficult to evaluate in natural systems, chiefly because it is difficult to track and measure the habitat preferences of vagile wild animals throughout their lives.

Common loons (*Gavia immer*) are attractive animals for investigation of habitat selection because of several life history traits (Piper et al., 2013). Loons are large, diving piscivores found on lakes throughout northern North America. Monogamous pairs occupy large territories, typically an entire small lake or part of a larger lake, and each resident pair member defends the territory against intruding “floaters” (young prebreeders) of the same sex, who seek to evict and replace the resident (Piper et al., 2000, 2006). Within each breeding season, chicks are reared entirely within a discrete lake or section of a large lake, which is usually distinct from other lakes used by other loon pairs for chick rearing. Consequently, habitat criteria between lakes can be quantified and compared for many environmental traits, facilitating studies on territory quality, habitat choice, and reproductive success (Piper et al., 2013, 2015). Loons also exhibit short‐range natal dispersal (Piper et al., 2012); thus, returning adults banded as juveniles (hereafter “ABJs”) can be identified and their habitat preferences tracked throughout life.

Young adult loons exhibit strong NHPI when recruiting into breeding territories, selecting lakes of similar pH and size to their natal lake (Piper et al., 2013). Fledglings, unlike adult settlers, must gain mass and develop flight muscles in preparation for their first southward migration, and thus might be expected to visit lakes that allow access to a rich food supply, rather than those promising high breeding success. Thus, in this study, we investigated physical and biotic aspects of lakes to determine whether juvenile loons after fledging express a form of habitat selection suited to their immediate fitness needs or exhibit NHPI similar to that of young adults settling on a first territory.

## 2 METHODS AND MATERIALS

### 2.1 Study area

Fieldwork was conducted within a 1,700 km^2^ study area in northern Wisconsin USA that lies chiefly in Oneida County, but includes portions of northern Lincoln County and southern Vilas County. This region is typified by temperate hardwood and conifer forests, and contains an abundance of glacial lakes that vary in size, hydrology, water chemistry, and prey community. Almost all lakes in this region experience a high degree anthropogenic activity in the form of angling and boating.

Adult loons migrate to the breeding grounds in spring, where pairs defend territories ranging from small lakes (<200 ha) to protected bays and regions of much larger lakes (>200 ha). Loons are apex consumers in these lakes, foraging largely on fishes including yellow perch (*Perca flavescens*), bluegill (*Lepomis macrochirus*), black crappie (*Pomoxis nigromaculatus*), and brown bullhead (*Ameiurus nebulosus*). Adults feed these fishes to their chicks, as well as minnows (Cyprinidae spp.), amphibians, crayfish, leeches, and snails (Merrill et al., 2005; Piper et al., 2013).

### 2.2 Field procedures

**FIGURE 1:**
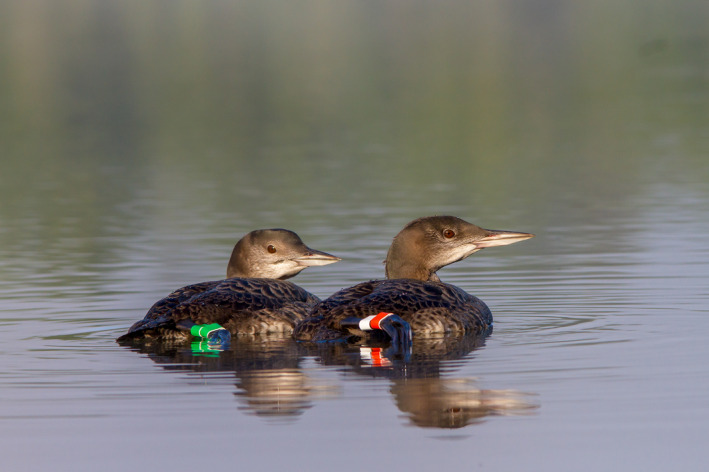
Two juvenile loons rest on Muskellunge Lake in Lincoln County, WI, showing color leg bands used for identification. (Photograph: Linda Grenzer)

We have systematically captured, banded, and observed loons in the study area since 1993 (Piper et al., 2015). During nocturnal capture, loons are spotlighted and netted from a small motorboat. We band each adult or chick 4 weeks and older with a metal USGS band and a unique combination of three plastic color bands (Figure 1; Gravoglas 2‐plex; Gravotech, Duluth GA). Loons are then weighed and released in their territories. Single observers in canoes make weekly visits to study lakes (chiefly between 0500 and 1300) to identify breeding pairs from leg bands, record breeding behavior, and document territorial intrusions by adults other than the breeding pair.

Juvenile loons capture most of their own food and become capable of flight by their 11th week (Merrill et al., 2005). At this age, they begin to leave their natal lake to forage on other lakes. In northern Wisconsin, this period begins in early September and continues until lakes freeze and juvenile migrate south, usually in November. For this study, we analyzed non‐natal lake visits of marked juveniles during September and October 2012–2015.

### 2.3 Basic lake variables

Our study lakes vary in physical, chemical, and biotic attributes. To understand loon responsiveness to these traits, we used a Basic Lake Features dataset. This dataset was derived from our long‐term study lakes, which have currently or historically supported breeding loons, and thus represent acceptable loon habitat. For the majority of these study lakes, we collected environmental data on (a) size or surface area (within study area: min. ~8 ha; max. ~1,400 ha), (b) maximum depth (min. ~3 m; max. 117 m), (c) lake shape (normalized as the perimeter divided by the square root of the surface area, min. 0.35 ; max. 3.9), (d) water clarity (estimated from the mean of secchi disk readings on different days in 2012; min. 0.75 m; max. 6.9 m), and (e) pH (estimated with a pH meter; YSI Pro Plus; YSI Incorporated; again the mean of readings on different days in 2012; min 4.7; max. 9.4).

### 2.4 Trophic classes

The trophic community of lakes in our study region varies widely (Emmons et al., 1999; Tonn & Magnuson, 1982); thus, we classified our study lakes also based on their trophic attributes to assess juvenile loon movements in the context of food availability and prey types. The Trophic Class dataset was based on studies of trophic lake communities in northern Wisconsin conducted by Rypel et al. (2019) and recognizes 12 types of lakes defined by discrete categorizations of trophic complexity (Complex => 4 sportfish species present; Simple =< 4 sportfish species), temperature (Warm or Cool), water clarity (Clear or Dark), and stratification dynamics (e.g., two‐story lakes exhibit significant vertical stratification such that both warm‐ and cold‐water fish communities are present within the same lake). As the Trophic Class dataset encompasses a wider spatial range than the Basic Lake Features dataset and contains water bodies that are not suitable for loons (i.e., rivers, trout ponds and lakes < 10 ha), we confined the Trophic Class dataset to overlap spatially with Loon Lake data and removed unsuitable water bodies.

### 2.5 Study design

**FIGURE 2:**
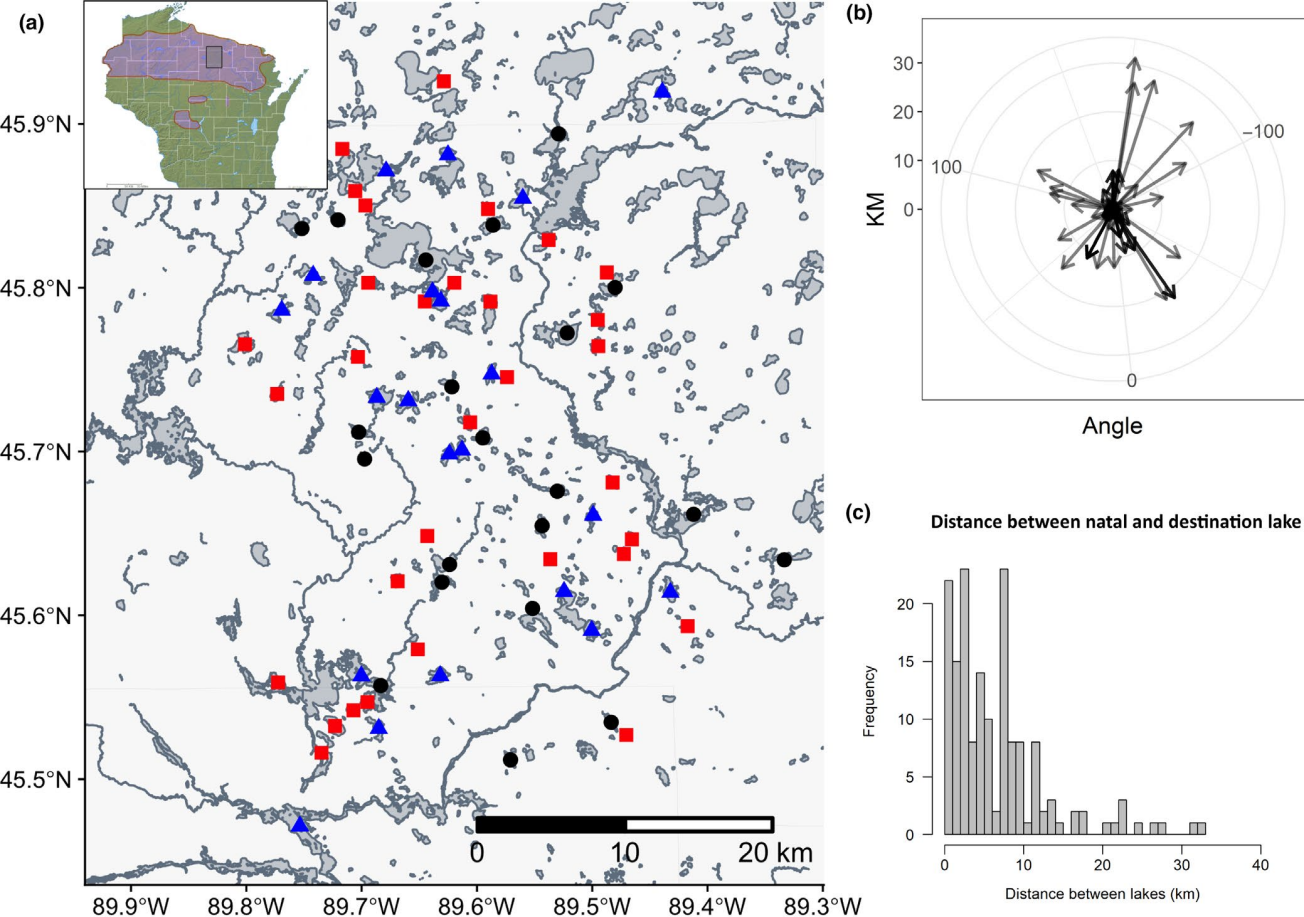
(a) Study area in northern Wisconsin, in which 120 lakes are monitored across approximately 3,500 km^2^. Shaded region in inset depicts breeding range of common loons in Wisconsin, and rectangle in inset depicts the approximate study region. Blue triangles depict lakes where nonresident juvenile loons visited in 2012–2015 (e.g., “Destination lakes”). Red squares depict lakes where the juvenile loons in the study originated from (“Natal lakes”). Black circles depict lakes that served as both destination and natal lakes. (b) Polar plot depicting the bearings and distances that juvenile loons traveled when leaving their natal lake. (c) Frequency distribution of the distances juvenile loons traveled between their natal and destination lakes (max = 33 km; mean = 7.5 km)

We define “natal” and “destination” lakes from the perspective of each juvenile loon in our dataset; for example, Lake A can serve as a natal lake for a juvenile that was hatched there, and it can also serve as a destination lake for a separate juvenile loon that visits. Natal and destination lakes were broadly distributed throughout the study region, with no apparent differences in spatial clustering between natal and destination lakes (Figure 2a). There was little indication of a directional bias in juvenile travel (Figure 2b). Juveniles travelled a mean distance of 5.7 km between natal and destination lakes (maximum distance: 32 km; Figure 2c).

To assess natal preference for lake attributes, we first identified all instances in which intruding chicks were observed on a non‐natal lake during September–October 2012–2015 (*n* = 167). We then filtered this initial data to include only instances where quantitative lake trait data for all five variables in the Basic Lake Features classification (lake size, lake depth, lake shape, pH, and water clarity) were available for both natal lake and destination lakes (*n* = 85 instances). These 85 observations included repeat sightings in which the same individual visited the same lake more than once on different dates (*n* = 34 repeat visits) and instances where an individual was observed visiting different lakes (*n* = 5 instances).

We applied separate Monte Carlo Randomization tests (Manly, 2006) to each lake variable to determine the likelihood of juvenile loons selecting lakes based on that attribute being more similar to their host lake than would be randomly expected. In this approach, the destination lake of each visiting juvenile was replaced with a randomly selected lake from the surrounding study area, sampling with replacement. The difference in lake variables between each natal and destination lake was computed, with the procedure repeated 10,000 times to produce an expected random distribution of means. This distribution was then compared to the observed mean between natal and destination lakes for each lake variable, and we determined statistical significance using Bonferroni‐corrected *α* values of 0.01.

Similarities between natal and destination lakes can be influenced by spatial autocorrelation, that is, lakes that are nearer to each other geographically are more likely to have similar traits than are lakes farther away. In a previous study on NHPI in young adults using the same study lakes, lake variables were not significantly spatially autocorrelated (Piper et al., 2013). As the average distance between natal and destination lakes exhibited by breeding adults could differ from that of foraging juveniles, however, we constructed semi‐variograms to estimate the approximate distance at which lake variables were spatially autocorrelated. For any significant randomization test results, we then examined how many data observations occurred within a distance that could have been influenced by spatial autocorrelation among variables. If the majority of lake visits occurred within the autocorrelation threshold, we used a second extremely conservative alternative approach to protect against the possibility of our results being produced by autocorrelated environmental variables. In this second approach, we constrained our randomization analysis by distance, by randomly resampling only those lakes that were within the spatial autocorrelation threshold. Randomly selecting from an autocorrelated distribution was highly conservative statistically, because a significant effect could only be detected given a very strong preference for similarity detectable despite spatial autocorrelation. That is, by spatially constraining our “random” pool of lakes to resample, we made it even more challenging to detect a preference for lake similarity.

Trophic comparisons between natal and destination lakes were assessed by comparing the observed frequencies of lake type visited against the expected frequency that each lake type should be visited, given their respective abundance within the study area. Expected distributions were created by calculating the number of loon‐friendly lakes (i.e., filtered for minimum lake size thresholds and excluding those lake classes that loons never inhabit, e.g., trout ponds) for each lake class type that were located within 40 km of all natal lakes. These expected proportions were then compared against the observed proportions using Goodness of Fit Chi‐Sq. tests.

Randomization tests, statistical analyses, and mapping were conducted in R Statistical Software (R Development Core Team, 2011), using the packages ggplot2, tidyverse, Leaflet, vegan, ggmap, and a customized script for randomization analyses.

## 3 RESULTS

We documented 43 juvenile loons making 85 lake visits, with these lake visits representing travel between 35 natal lakes and 27 destination lakes. The distribution of lake trait data for natal lakes did not differ from the distribution of data within all other study lakes used in the Basic Lake Features dataset (Figure S1). However, destination lakes overall were larger and of higher pH than natal lakes (Figure S1; Table 1).

**TABLE 1:** Comparison of lake traits between Natal (*n* = 35) and destination (*n* = 27) lakes; Welch Two‐sample *t* test using Bonferonni‐adjusted *a* (0.05/5 = 0.01)

Lake traits	Natal x̅	Destination x̅	*Df*	*f*	*p*‐value
Lake depth	28.69	32	57.5	0.82	0.42
Lake size	55.64	176.55	31.1	3.38	0.002
Lake roundness	0.61	0.70	55.03	2.64	0.011
Lake pH	7.12	7.82	61	3.68	0.0005
Secchi depth	10.08	9.14	46.1	−0.81	0.42

**FIGURE 3:**
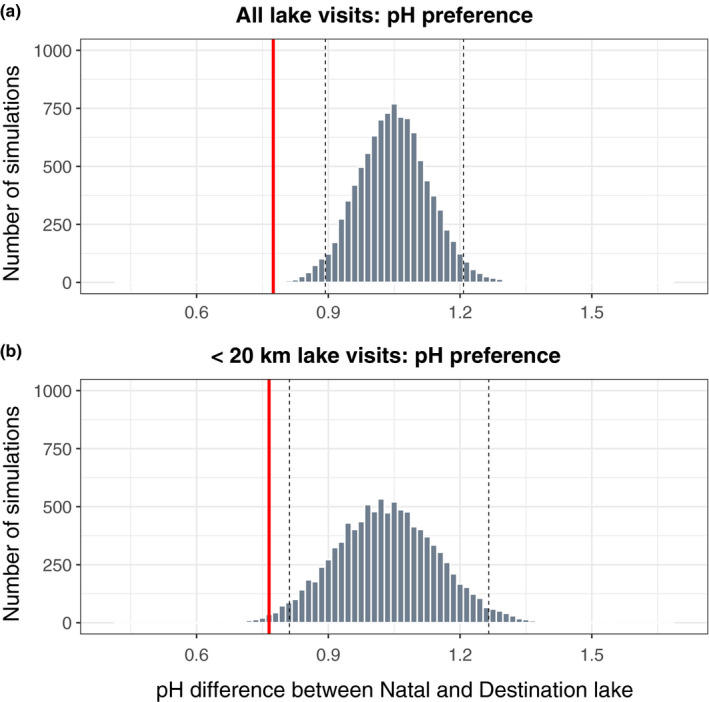
Randomization tests depicting the observed mean differences in lake variables between natal and destination lakes (solid black line) and the distribution of mean differences computed from a resampling simulation (dotted lines depict 95% confidence intervals). (a) Juvenile loons (*n* = 85) showed a significant tendency to visit lakes that are more similar in pH to their natal lake than would be randomly expected. (b) When data are subset into only those loon visits occurring within distances potentially affected by spatial autocorrelation (22 km; *n* = 77), juvenile loons exhibit a reduced yet significant likelihood of visiting lakes significantly more similar in lake pH than would be randomly expected

Juvenile loons exhibited a strong preference for foraging lakes that resemble their natal lake in pH (Figure 3a; *p* < 0.0001). However, pH values, unlike all other lake variables measured, were spatially autocorrelated within approximately 20 km with a significant anisotropic gradient occurring along a Southwest‐Northeast bearing (Figure S2). When we carried out a second more conservative randomization analysis, which accounted for such spatial autocorrelation by constraining random resampling of lakes to include only those lakes within 20 km of the natal lake (*n* = 72), we also detected a significant preference for juveniles to visit lakes of similar pH to their natal lake (Figure 3b, *p* = 0.009). In contrast, juvenile loons did not visit destination lakes similar to their natal lake in size (*p* = 0.20), shape (*p* = 0.60), maximum depth (*p* = 0.81), or water clarity (*p* = 0.10).

**FIGURE 4:**
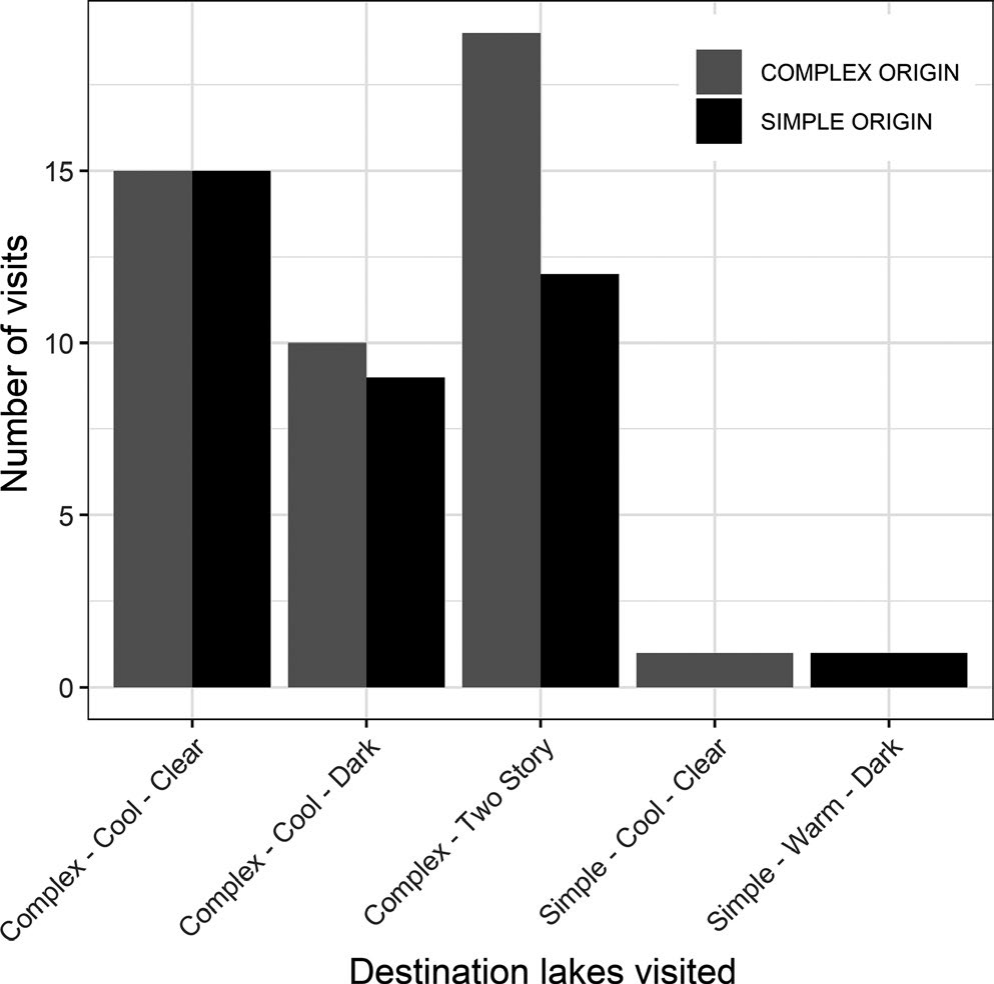
Distribution of lake visits from juvenile loons raised in either Complex lakes (light gray) or Simple lakes (dark gray). Irrespective of natal lake origin, all juveniles visits exhibited a significant preference for visiting destination lakes of Complex classes (Complex Lakes: *X* ^2^ = 81.93. *df* = 5, *p* < 0.0001)

**FIGURE 5:**
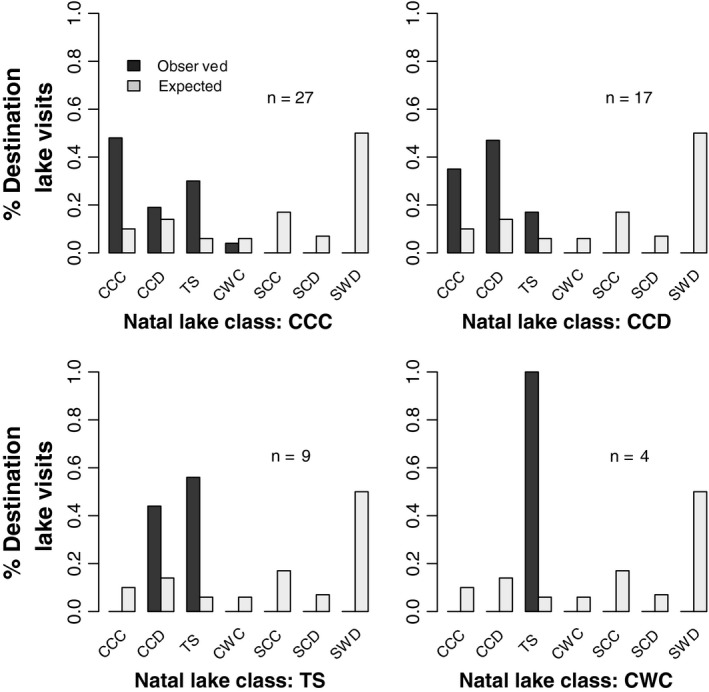
The proportion of juvenile loons visiting different lake types relative to their own natal lake class. Juvenile loons originating from Cool‐Complex‐Clear (CCC) lakes, Cool‐Complex‐Dark (CCD) lakes and Two‐Story (TS) lakes visited their own lake classes in greater proportions than would be expected given the background distribution of lake classes (CCC: *X* ^2^ = 81.93. *df* = 6, *p* < 0.0001; CCD: *X* ^2^ = 39.81, *df* = 6, *p* < 0.0001; TS: *X* ^2^ = 49.96, *df* = 6, *p* < 0.0001). Other designations include Complex‐Warm‐Clear (CWC) lakes, Simple‐Cool‐Clear (SCC) lakes, Simple‐Cool‐Dark (SCD) lakes, and Simple‐Warm‐Dark (SWD) lakes

Juvenile loons showed a strong tendency to visit large and trophically complex lakes (Figure 4; Complex Lakes: *X* ^2^ = 81.93. *df* = 5, *p* < 0.0001). Juvenile loons raised on simple lakes (i.e., trophically constrained and often smaller in size) always visited larger and more trophically complex lakes, regardless of clear–dark or warm–cold designations. Juvenile loons raised on complex lakes, however, showed a significant tendency to visit the same class of complex lake on which they were raised (Figure 5: CCC: *X* ^2^ = 81.93. *df* = 6, *p* < 0.0001; CCD: *X* ^2^ = 39.81, *df* = 6, *p* < 0.0001; TS: *X* ^2^ = 49.96, *df* = 6, *p* < 0.0001).

## 4 DISCUSSION

### 4.1 Variation in natal habitat preference and life stage

NHPI has been previously documented in young adult loons in our study area (Piper et al., 2012, 2013), but here we show here a new age‐dependent pattern in NHPI. Whereas young adults prefer to settle on breeding lakes similar in both pH and size to their natal lake (Piper et al., 2013), juveniles preferentially visit large lakes and only express NHPI in the context of lake pH. This difference between young adult and juvenile loon NHPI patterns likely reflects life‐stage differences in energetic needs and fitness.

Young adults appear to select breeding territories based on reproductive fitness consequences, evaluating lakes on the basis of cost‐benefit trade‐offs between food availability and territorial defense (Piper et al., 2013). This trade‐off is influenced by lake size; smaller lakes may be more easily defended and lead to a reduced threat of eviction, whereas larger lakes might provide more food yet incur more territorial disputes and threat of eviction. Juveniles, in contrast, are more likely to select lakes based on their immediate energetic needs. After fledging, juveniles must gain weight and prepare for the physiological challenge of their first fall migration. Thus, regardless of lake origin, juvenile loons primarily visited large, complex lakes that are more likely to provide abundant food (Parks & Rypel, 2018), in effect showing no natal preference for lake size.

The differences between juvenile and young adult NHPI may also be driven by seasonal differences in prey availability across lakes. For example, some lakes with abundant food resources that seem good for nesting in spring may become resource‐depleted by fall due to predation and recreational fishing (Rudstam & Magnuson, 1985). Young adult loons recruit into their habitats in early May, as soon as the winter ice recedes (Piper et al., 2006, 2015). This time period precedes spring productivity blooms and summer growth in young‐of‐the‐year fish cohorts; therefore, resource limitation may not be an immediate constraint for territory‐seeking adults. Over the course of a summer, however, a loon family exerts considerable predation pressure; Barr (1996) estimated that a family of two adult loons and two chicks can consume approximately 423 kg of prey during the 5.5 month breeding period, a considerable prey reduction in a small lake. Thus, for juveniles needing access to abundant food in the fall, prey densities in smaller lakes may no longer be sufficient.

### 4.2 Lake pH as a habitat feature

In our study system, pH is primarily a static variable; the lakes of northern Wisconsin exhibit low alkalinity and pH that reflect the glacial origins of the bedrock and watershed, and the pH range of these lakes do not vary significantly over time (Lillie & Mason, 1983). However, regional differences in soil composition and water flow (e.g., seepage vs. drainage lakes) ensure that moderate pH differences can occur between nearby lakes (Lillie & Mason, 1983). Thus, in our study system, the strong preference of both young adult and juvenile loons to visit lakes with a pH similar to their natal lake suggests that pH may partly function as a temporally consistent indicator of habitat type.

If pH remains regionally and temporally consistent across lakes, then adults and juveniles may both be seeking familiar foraging habitats by responding to complex relationships between lake pH, water clarity, food availability, and foraging efficacy among specific lake types. While the exact nature of such a mechanism remains unclear, lake pH is known to vary with physical and biotic lake variables that could physically influence visual conditions or lake habitat. For example, seepage lakes, which exhibit a significantly lower pH that drainage lakes, also have generally clearer water with less eutrophication than drainage lakes (Lillie & Mason, 1983). Lake pH can also influence biotic variables that physically structure lake habitats, particularly via aquatic vegetation. A review of freshwater systems reported a strong influence of lake pH on the diversity and abundance of aquatic vegetation (Lacoul & Freedman, 2006), and vegetation provides important habitat for locally important loon prey species such as crappies and bluegill (Valley et al., 2004).

For loons, the most important role of pH may be in its significant association with specific food web communities. In Ontario lakes, for example, Matuszek and Beggs (1988) found that among acidic lakes (pH < 6.0), pH explained 21% of the variation in fish diversity. Among important prey species in north Wisconsin lakes, Yellow Perch and sunfish tolerate acidic water whereas many cyprinids do not (Rahel, 1986; Rahel & Magnuson, 1983). Thus, the tendency of juvenile loons to visit large complex lakes most similar to their natal lake class may also reflect a preference for familiar food webs associated with specific pH cues, which could then lead to improved foraging efficacy. In short, pH represents a relatively persistent feature that loons could use to track habitat type over space.

### 4.3 The importance of food web familiarity and prey accessibility to juvenile loons

Most juvenile animals lack the experience to forage as efficiently as adults, and may compensate for this inefficiency by foraging longer, consuming poorer quality prey items, or exhibiting different types of foraging behaviors (Wunderle, 1991). For many birds, juvenile foraging limitations are further exacerbated by the need to acquire sufficient mass to support fall migration. Loons, in particular, have extremely high wing‐loading (body mass divided by wing area), and juvenile loons face severe energetic constraints that must be prioritized for short‐term survival and successful migration. Thus, the movement pattern we observed in young loons—their tendency to visit large, complex lakes of a similar natal pH—is likely in response to regional differences in prey abundance and habitat familiarity across lakes. If the nascent prey capture abilities of juvenile loons are attuned to finding specific prey types in specific lake conditions, and these conditions are linked to pH variability, juvenile preference for habitats similar in pH to their natal lake is clearly advantageous.

Juvenile loon preferences for large lakes are reflected in how prey resources vary across the lake classes used in this study (Rypel et al., 2019). The primary predictor of overall fish abundance is lake size; simple lakes are uniformly small and lack trophic diversity, whereas large complex lakes have high trophic diversity with maximum trophic levels of 4–5 (Rypel et al., 2019). While abundances of small fishes can be high in small lakes, they lack large calorically dense meals for loons, which may explain their limited preference for these lakes. In contrast, large two‐story lakes hold cold and well‐oxygenated deep‐water habitats that support populations of pelagic prey fishes like cisco and whitefish (Parks & Rypel, 2018; Rudstam & Magnuson, 1985). These fishes are calorically dense, and support rigorous growth rates and healthy predator populations in lakes (Jacobson, 1992; Matuszek et al., 1990). Though the area of large lakes appears vast, ironically, these fishes may be easy to predate because of their schooling tendencies, and depth limitations in the summer months when most pelagic fishes are constrained to relatively narrow metalimnetic (thin, cool and oxygenated) layers of the water column (Ahrenstorff et al., 2013; Hansen & Beauchamp, 2015). Thus, in the preference of nearly all juvenile loons for large, complex lakes, they may first be seeking locations with the highest likelihood of finding an accessible source of these lipid‐rich prey species.

## 5 CONCLUSIONS

Here, we show that study of the juvenile stage can provide useful insights into the development and maintenance of behavioral processes, such as NHPI. Such studies are uncommon, however, as long‐term datasets that facilitate careful hypothesis testing on juvenile behavior are rare.

This is unfortunate, as there are proven adaptive advantages for juveniles to imprint from natal conditions and benefit from reusing natal habitat later in life (Gerlach et al., 2007; Grassman, 1993). Consequently, juveniles offer a mechanistic window into the behavioral development of NHPI behaviors expressed in adults. As this study shows, juveniles may, in effect, ignore or devalue their natal habitat preference during their first few months of life, choosing to focus on immediate survival, rather than eventual reproductive success.

The contrast we report between juvenile and young adult natal habitat selection shows that environmental preferences shift over time, even within the same habitat. This result highlights the need to incorporate an understanding of multiple life stages in ongoing policy considerations regarding loon conservation and fisheries management. Loon populations have declined significantly at our study site over the last three decades, with juveniles exhibiting reduced survival and a decline in mass (Piper et al., 2020). As temperate habitats experience climate change, clarifying the habitat‐life stage interactions of this iconic northern species might help us mitigate future shifts in lake ecology and habitat quality. For the loons of northern Wisconsin, we establish here a new ecological template for future studies on the interactions between trophic patterns, behavior, and demography.

## CONFLICT OF INTEREST

None declared.

## AUTHOR CONTRIBUTIONS

**Brian A. Hoover:** Analysis (lead); investigation (equal); methodology (lead); validation (lead); visualization (lead); writing – original draft (lead); writing – review and editing (equal). **Kristin Brunk:** Data curation (equal); writing – review and editing (equal). **Gabriella Jukkala:** Data curation (equal); writing – review and editing (equal). **Nathan Banfield:** Data curation (equal); writing – review and editing (equal). **Andrew L. Rypel:** Methodology (supporting); resources (supporting); validation (supporting); writing – original draft (supporting); writing – review and editing (equal). **Walter Piper:** Conceptualization (lead); data curation (equal); funding acquisition (lead); investigation (equal); methodology (supporting); project administration (lead); resources (lead); supervision (lead); validation (supporting); visualization (supporting); writing – original draft (supporting); writing – review and editing (equal).

## Supporting information

Figure S1‐2

## Data Availability

The datasets used during the current study and corresponding R scripts are available in the “Loon Project Database” repository through Chapman University Digital Commons (https://digitalcommons.chapman.edu/sees_data/3/) and available from Dryad (https://doi.org/10.5061/dryad.18931zct3).
