# Supplementary material for: Early evidence of natal‐habitat preference: Juvenile loons feed on natal‐like lakes after fledging
Source: Ecol Evol. 2020 Dec 28;11(3):1310–9. doi: 10.1002/ece3.7134 (PMC7863666; doi:10.1002/ece3.7134)
Supplement: Supplementary file 1 — Figure S1‐2 [file ECE3-11-1310-s001.docx]

**Supplemental Figures**

**
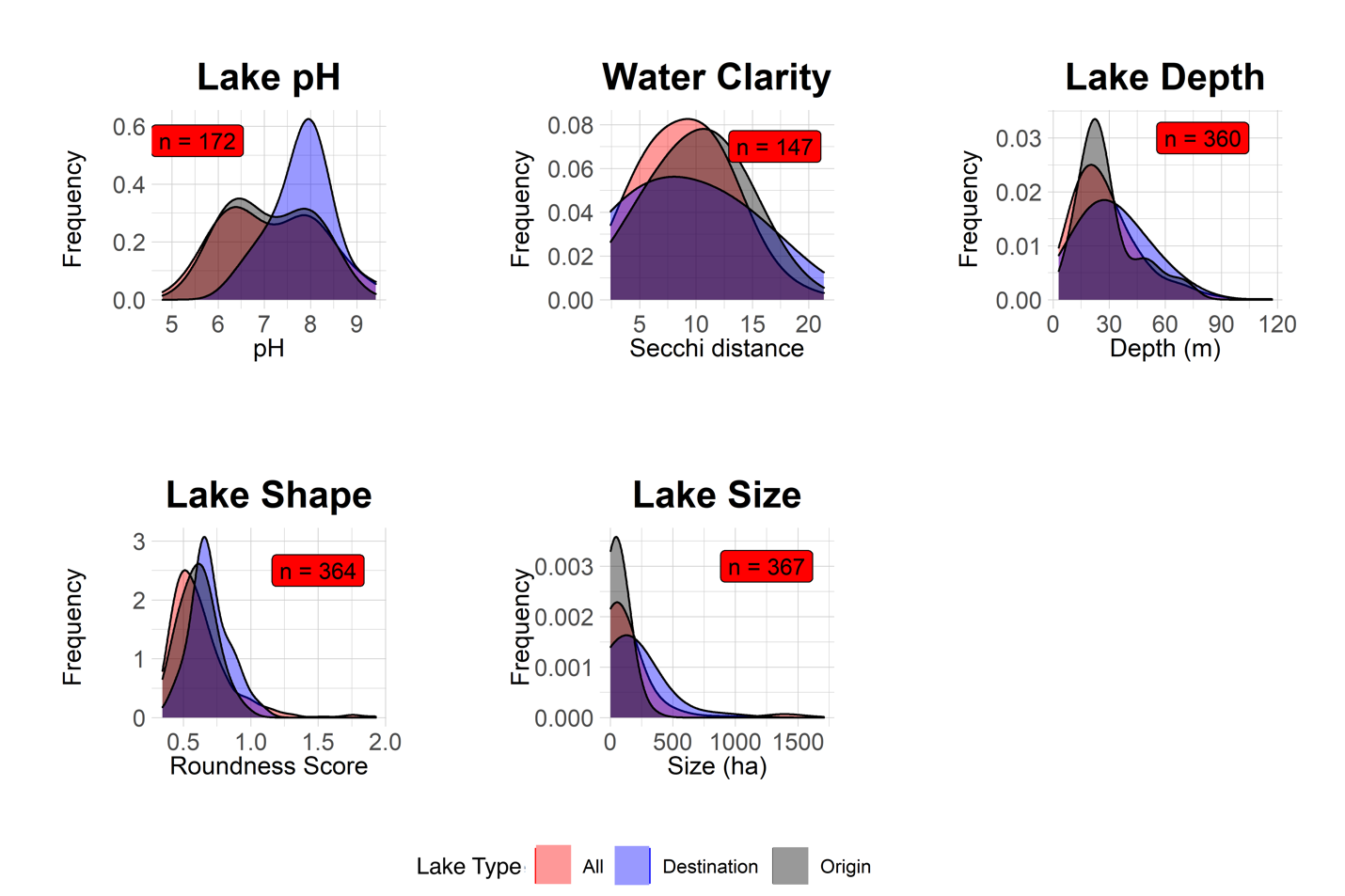
**

**Fig 1:** Density plots depicting the distribution of data for each lake variable used in randomization test analyses, comparing Destination Lakes (n = 27), natal lakes (n = 35), and all other lakes in our study region with observed loon pairs (sample sizes in red).

**
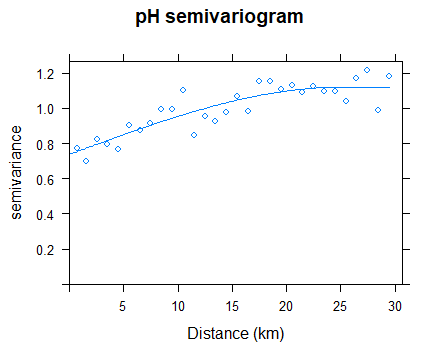
**

**Fig 2a:** Semi-variogram showing 20-22 km spatial autocorrelation threshold among lakes assessed in the study..


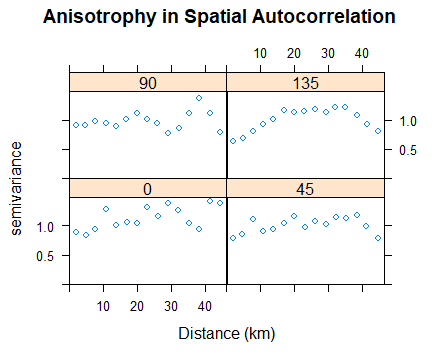


**Fig 2b:** Semi-variograms showing anisotropic directional effects of spatial autocorrelation in lake pH values. Lake pH values are largely autocorrelated along North-West to South-East gradients, suggesting spatial autocorrelation effects are not universal.
